# Can Dual Infrared–Visual Thermography Provide a More Reliable Diagnosis of Perforator Veins and Reflux Severity?

**DOI:** 10.3390/jcm12227085

**Published:** 2023-11-14

**Authors:** Marcelo Pastor Almada Dávalos, Marcos Leal Brioschi, Samir Ezequiel da Rosa, Gabriel Carneiro Brioschi, Eduardo Borba Neves

**Affiliations:** 1 Argentine Medical Association, National University of La Plata (FMUNLP), La Plata B1900, Argentina; 2 Neurology Department, Hospital das Clínicas, São Paulo University, Sao Paulo 05508-220, Brazil; 3 Brazilian Army Research Institute of Physical Fitness (IPCFEx), Rio de Janeiro 22291-090, Brazil; 4 Greenways Academy School, St. Louis, MI 63141, USA; gabrielbrioschi1@gmail.com; 5 Graduate Program in Biomedical Engineering, Federal Technological University of Paraná (UTFPR), Curitiba 80230-901, Brazil; neveseb@gmail.com

**Keywords:** perforators veins, reflux severity, thermography, vascular dysfunction, venous doppler

## Abstract

The accurate identification of perforator veins (PV) in asymptomatic veins that do not meet the criteria established by venous Doppler (VD) is a complex challenge, considered the gold standard in diagnosis, and is operator-dependent. This study explored the potential of dual infrared–visual thermography (IRVT) to identify PV in 99 patients aged 29 to 80 years. IRVT was conducted using a high-definition hyperspectral visual–infrared sensor. The temperature difference (ΔT) between maximum temperature (Tmax) and minimum temperature (Tmin) within the region of interest (ROI) served as an indicator for assessing vascular dysfunction severity. Comparative analysis was performed with VD results obtained using a Doppler ultrasound unit equipped with a 7.5 MHz linear transducer. Significant statistical differences (*p* < 0.05) in ΔT (Tmax−Tmin) were observed among PV sites categorized by reflux severity: no reflux (ΔT = 1.2 °C), mild reflux (ΔT = 1.8 °C), moderate reflux (ΔT = 2.9 °C), and severe reflux (ΔT = 3.6 °C). This study concludes that IRVT effectively distinguishes varying degrees of vascular reflux severity. IRVT shows promise as a non-invasive, radiation-free tool to enhance PV identification, especially in challenging cases, potentially improving patient outcomes and healthcare management. Further research is required to validate and refine its diagnostic utility.

## 1. Introduction

Chronic venous insufficiency (CVI) is a prevalent lower-limb condition with varying occurrence rates globally. Although trophic alterations and venous ulcers are observed in 3–13% and 1–2.7% of cases, respectively, varicose veins affect 1 in 4 adults, escalating to 70% when considering smaller vessels. Perforating vein (PV) dysfunction, despite a 30% incidence in total venous disease, is often neglected in diagnosis. Its significance is underscored by its association with clinical severity in the CEAP classification. Incompetent PVs play a crucial role in the pathogenesis of venous diseases, necessitating timely identification for effective prevention and treatment [1,2,3,4,5,6,7,8,9].

Although venous Doppler (VD) evaluation is considered the “gold standard” for identifying venous disease [9], the application of dual infrared–visual thermography (IRVT) has shown promise in detecting visual with temperature changes in areas corresponding to dysfunctional PVs, aiding in diagnosis and monitoring of disease progression by some authors [8,9,10].

Infrared Thermography (IRT) is a contact-free, radiation-based diagnostic method that rapidly and safely studies the heat emanating from the skin body [10,11,12]. Notably, its applications extend beyond chronic venous insufficiency (CVI), encompassing diverse vascular fields such as peripheral arterial disease (PAD) [11,12]. Recent studies, like Zenunaj et al.’s investigation of symptomatic PAD patients undergoing endovascular revascularization [11] and Piva et al.’s systematic review of IRT’s utility in assessing foot perfusion in relation to various interventions for PAD [12], underscore its potential as a valuable tool for clinical evaluation and treatment efficacy assessment in vascular disorders.

This thermal imaging analysis can prove advantageous as it can detect, without contact, most perforator veins (PVs) and promises to assist in identifying reflux, therefore optimizing scanning efficiency. IRTV can show an area 3 to 5 times larger with an altered temperature gradient of 19. Furthermore, this diagnostic method appears to be an ideal complement to VD due to its portability, immediate results, and high sensitivity and specificity in identifying symptomatic or asymptomatic insufficient PVs, with or without the use of sonographic criteria for pathological inclusion [10,11,12,13,14]. This technique has been used to identify PVs since the 70s, with promising results.17 The primary objective of this study was to explore the association between skin temperature, assessed through dual infrared–visual thermography (IRVT), and perforator veins (PV) severity, as determined by clinical examination and venous Doppler (VD). This investigation sought to ascertain whether dual IRVT could effectively distinguish varying degrees of vascular reflux severity, ultimately enhancing PV identification and offering potential improvements in healthcare management.

## 2. Materials and Methods

### 2.1. Ethical Procedures

This study was authorized by the Ethics Committee General Hospital of Las Heras (HGLH) (Buenos Aires, Argentina), with number 030509-1, following the recommendations of the Helsinki Declaration [15]. All subjects signed the Informed Consent Form before starting the procedures.

### 2.2. Sample

This is a non-experimental, descriptive, retrospective, and cross-sectional study conducted between July 2020 and July 2022 at the General Hospital of Las Heras (HGLH) (Buenos Aires, Argentina). The sample comprised 99 subjects (50 men and 49 women), with a mean age of 56.6 ± 13.4 (range 29–80 years), randomly selected from patients treated at the Phlebology and Lymphology Service of HGLH. Each leg was considered separately; thus, there were 2 sets of 198 limbs to be compared.

The inclusion criteria were patients presenting with clinical symptoms of venous disease, confirmed by venous Doppler ultrasound examination. Exclusion criteria were as follows: previous vein treatment surgery or ablation, active deep vein thrombosis (up to 1 year), acute thrombophlebitis, lipedema, lymphedema, erysipelas, dermatitis, post-thrombotic syndrome, venotonic/hemorreologic and anti-inflammatory medication use, under 18 years old, inability to understand simple commands, unable to stand, distal arterial disease (PAD), fever, abscess, refusal to participate in the study, and patients living outside the city limits (excluded due to distance). Patients with previous sclerotherapy for cosmetic reasons were not an exclusion criterion.

### 2.3. Assessment Instruments

The study volunteers were assessed and categorized using the CEAP classification system, first established in 1994 and revised in 2020 [5,16], as presented in Table 1.

The VD examination was performed using the Z5 portable Color Doppler digital ultrasound system (Mindray, Shenzhen, China) with a 7.5 MHz linear transducer, following the protocol established by the Sociedad Argentina de Ultrasonografía en Medicina y Biología (SAUMB). PV reflux was classified according to duration and diameter as follows: mild refluxes from 0.5 to 1 s, moderate reflux from >1 to < 4 s, and severe reflux >4 s. The diameter was considered insufficient if the PV was greater than 3.5 mm at the fascial level [5,11,12,13,17].

The thermal infrared images were captured with the exposed leg with the patient standing; patients were not permitted to lie down prior to the image creation, and walking was encouraged but not controlled. The IRVT dual imaging was conducted using a visual–infrared hyperspectral sensor (E8, FLIR Systems, Inc., Wilsonville, OR, USA) with a high infrared long-wave definition of 320 × 240 (76,800 pixels), thermal sensitivity as less than 60 mK (0.06 °C) at 30 °C, visual image resolution of 640 × 480 pixels (307,200 pixels), frame rate of 30 Hz, and a field of view of 45° × 34°. The sensor was calibrated for evaluation of the temperature range of the human body (medical version). The test was performed in a climate-controlled room at 23 °C, with humidity below 60% and air velocity lower than 0.2 m/s. The patient rested for 15 min with the study area uncovered to achieve thermalization. The camera’s emissivity was set to 0.98. Patients were advised to refrain from shaving, exercising, taking a hot shower, or using elastic compressions (compression stockings-elastic bandages) or splints up to 2 h before the study, following the recommendations of the Brazilian Association of Medical Thermology (ABRATERM) [13,14].

The images were captured using a tripod-mounted camera positioned at a distance of 1–2 m (depending on the limb size) and directed toward the patient, who was lying in a horizontal position. Three views of each leg, including anteromedial, anterolateral, and posterior, were evaluated. To demonstrate PV dysfunction, which was previously assessed by VD, a 10-s Valsalva maneuver, knee flexion–extension, or leg dorsiflexion was performed depending on the patient’s physical condition.

**Figure 1:**
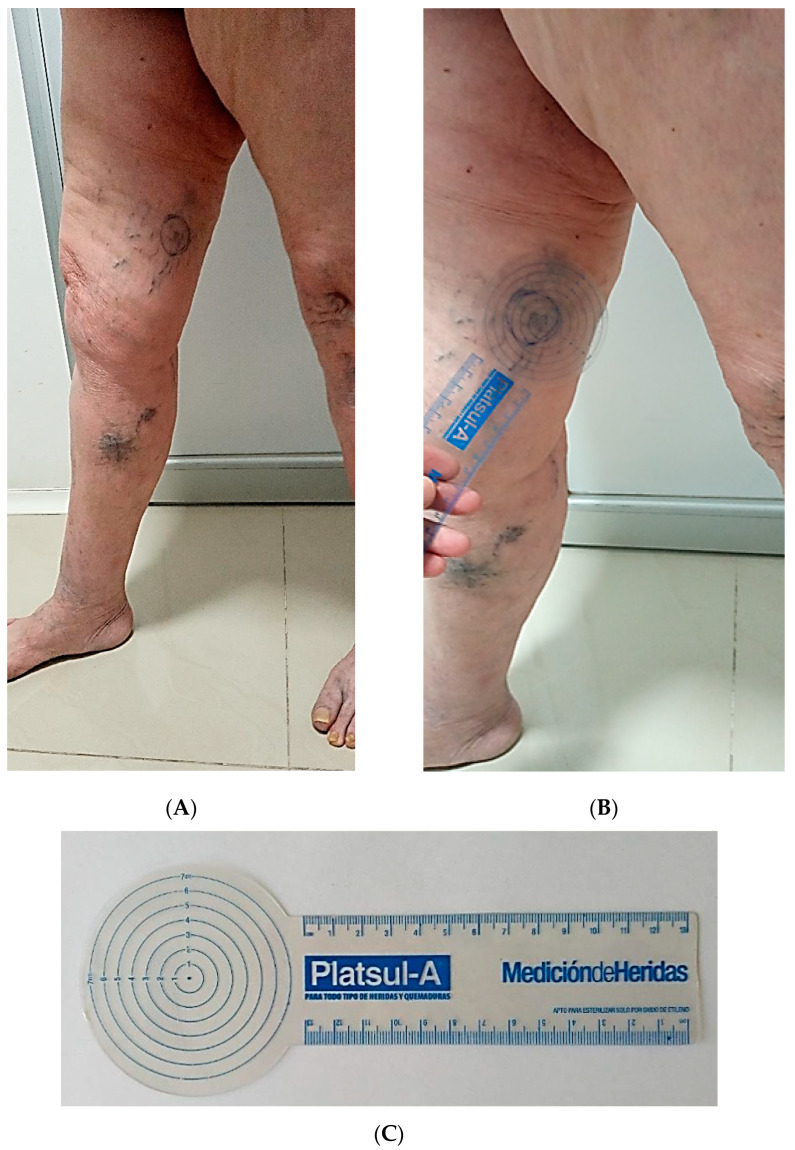
(**A**) Anatomical outlet site of Hunter’s perforator vein in a woman with CEAP 4 identified by VD. (**B**) The circle indicates the region of interest (4.0 cm^2^). (**C**) Instrument for measuring the anatomical region of interest. CEAP: Clinical-Etiological-Anatomical-Pathophysiological classification system. VD: venous Doppler.

**Figure 2:**
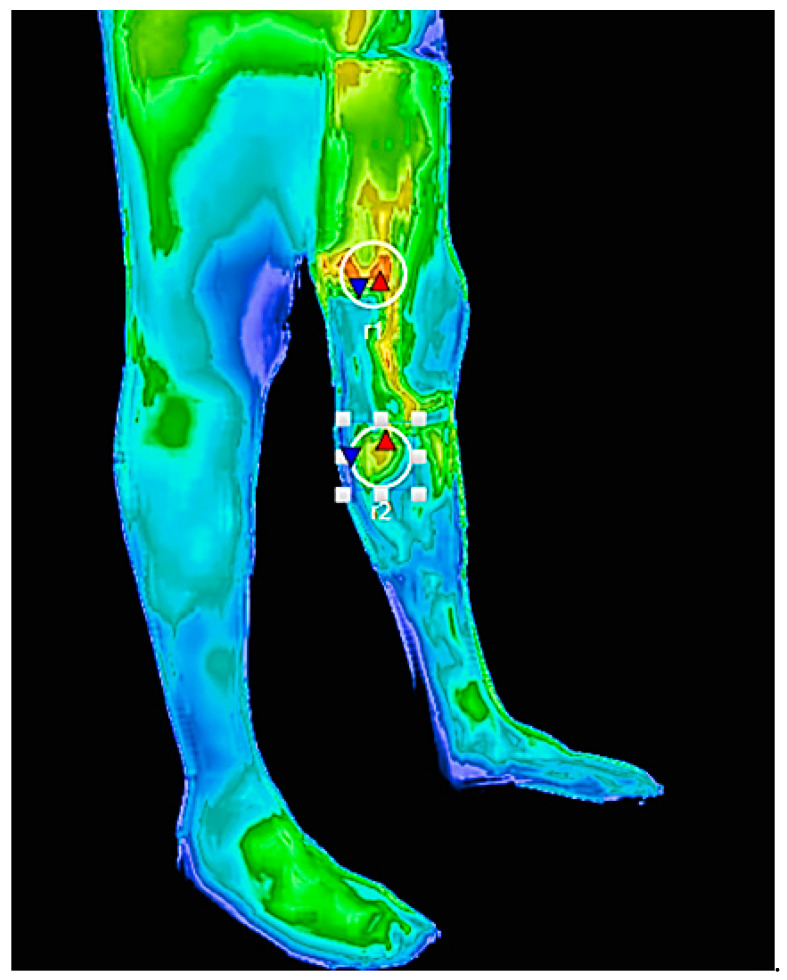
Thermal imaging assessment of a 50-year-old man with PV in the thigh and left leg, CEAP class 3. The ROIs, marked with a pen in an area of 4.0 cm^2^, were Hunter’s ROI r1 PV with moderate reflux (ΔT 4.26 °C) and Boyd’s ROI r2 PV with severe reflux (ΔT 3.33 °C). ROI r1 was selected for the study due to its higher thermal difference (temperature scale: min 26 °C, max 36 °C). The red triangles indicate the perforator location, while the blue triangles mark a nearby normal skin perfusion reference area.

The images were acquired using a device that measures the lesion diameter within an area previously delimited by a circular marker corresponding to the anatomical outlet of the PV, known as the imaginary Linton’s line [6]. The limit of 4.0 cm^2^ was considered in this area (Figure 1). The PVs were described using eponyms. The highest temperature (Tmax) and the lowest temperature (Tmin) were recorded in an oval-shaped thermal region of interest (ROI) of 4.0 cm^2^. A local delta T (ΔT) value was calculated from the difference between Tmax and Tmin of the ROI. One PV ROI per patient was selected, and if a patient had more than one ROI, the one with the greatest thermal difference was chosen (Figure 2). The images were analyzed using the VisionFy^®^ medical software v.1.2.1 (Thermofy^®^, São Paulo, Brazil), with a temperature range of 26.0 to 36.0 °C represented by a graduated color map scale. By utilizing software, IRTV can be integrated into the patient’s electronic medical record system, therefore facilitating data recording and analysis. The potential to incorporate IRTV into digital health platforms optimized the collection and storage of pertinent clinical information.

### 2.4. Statistical Analysis

The data collected was analyzed statistically using JAMOVI Software version 2.3.13. The Shapiro–Wilk test was conducted to assess the normality of the variables, and therefore, a parametric approach was adopted. Descriptive statistics were used to present the results of the clinical variables of CEAP classification, reflux, and temperatures evaluated by IT. Measurements for each leg were nested within the individual. Analysis of variance (ANOVA) with Tukey’s post hoc test was conducted to assess the variation in temperature (delta T or ΔT) among the reflux categories identified by VD. The level of statistical significance was set at 95% (α = 0.05).

## 3. Results

Out of the 99 patients included in this study, a smaller proportion was classified as CEAP 1–2 (30%), while the majority was classified as CEAP 3–4 (70%). Among them, 60.4% corresponded to the Hunter (distal medial thigh), Boyd (paratibial, medial leg 10 cm below the knee), Sherman (paratibial, medial leg 24 cm above the plantar surface), and upper fibular PVs. The latter is the least studied and often overlooked by specialists in vascular medicine due to its low prevalence and association with few symptoms of PV (Table 2).

**Figure 3:**
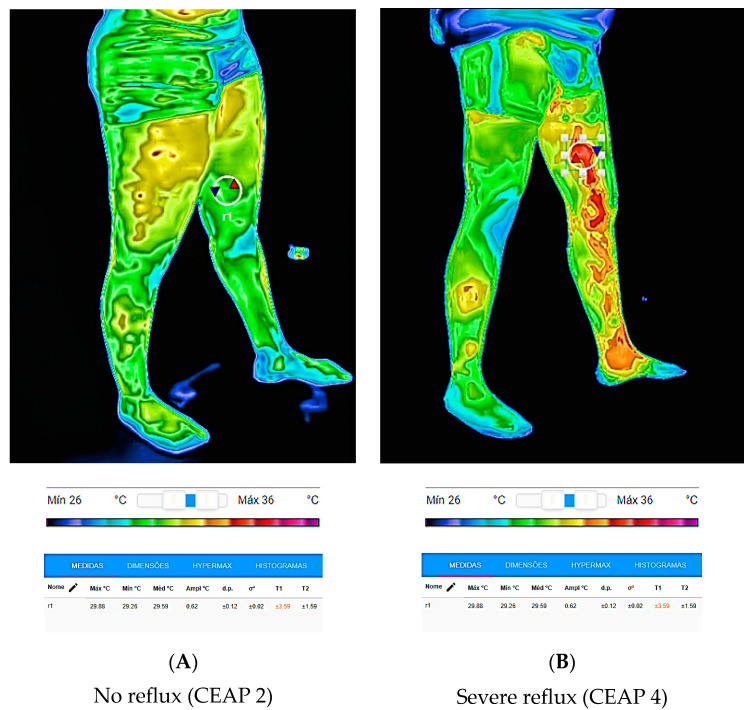
Thermal values of the perforator veins according to the degree of reflux. (**A**) A 37-year-old woman without Hunter PV reflux (ROI r1 ΔT 0.62 °C), (**B**) A 48-year-old man with severe Hunter PV reflux (ROI r1 ΔT 4.66 °C). CEAP: Clinical-Etiological-Anatomical-Pathophysiological classification system.

Thermal values were measured in patients with and without venous reflux, classified as mild, moderate, or severe, as shown in Figure 3.

**Figure 4:**
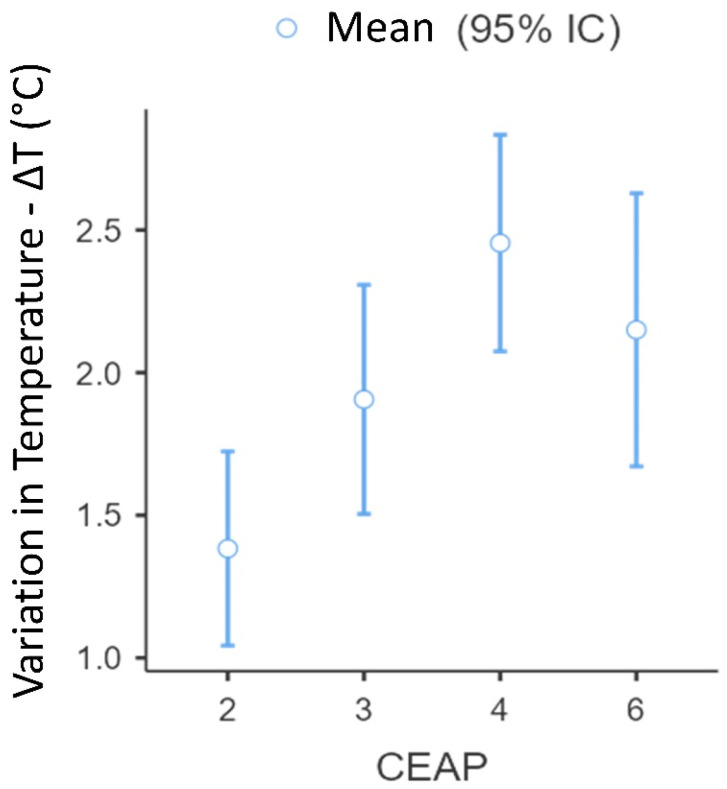
Variation in temperature delta according to CEAP classification (*n* = 99). A significant difference was found between patients classified as CEAP 2 and 4 (*p* = 0.014). CEAP: Clinical-Etiological-Anatomical-Pathophysiological classification system.

The results presented in Table 3 demonstrate the thermographic evaluation according to the CEAP classification of veins in the lower limbs (*n* = 99). To analyze the temperature delta difference between CEAP groups, cases with CEAP 1 (case 1) and 5 (case 2) were removed from the analysis due to the limited number of cases. A one-way ANOVA was calculated to determine the temperature delta (Tmax−Tmin) between groups classified by CEAP level, and significant differences were observed in the temperature deltas among the groups (F = 6.843 and *p* < 0.001), as shown in Figure 4.

In Figure 4, the temperature delta variation is presented according to the CEAP classification. A statistically significant difference was found only between patients classified as CEAP 2 and 4 (*p* = 0.014).

**Figure 5:**
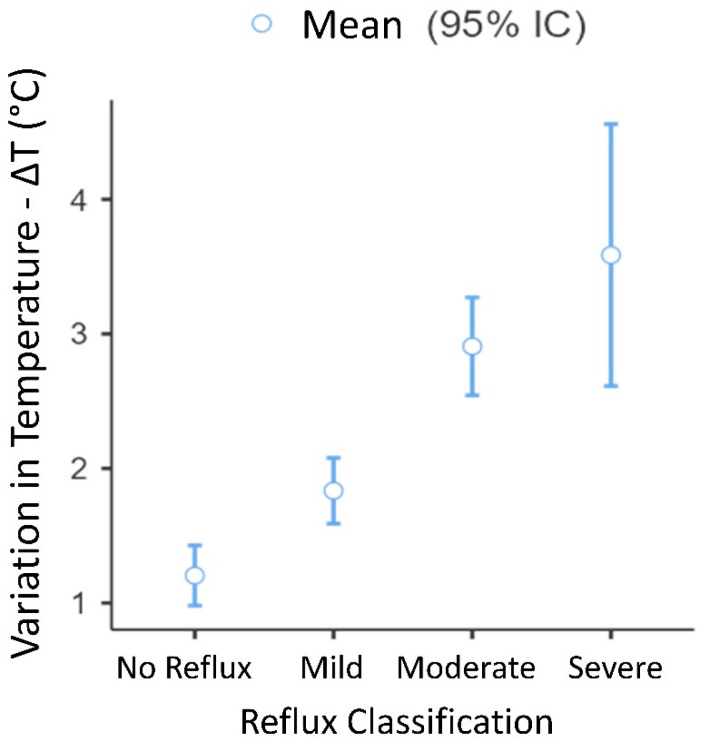
Temperature delta variation (ΔT) according to reflux classification (*n* = 99). Exact values: No reflux 1.18 ± 0.531, Mild 1.83 ± 0.774, Moderate 2.92 ± 0.929, and Severe 3.59 ± 1.053. Significant differences were found between all categories (*p* < 0.05), except between the “moderate” and “severe” categories.

Table 4 displays the findings of the thermographic assessment based on the classification of venous reflux in the lower limbs. A one-way ANOVA was performed to analyze the temperature delta (Tmax−Tmin) among groups categorized by the level of reflux identified in the VD. The ANOVA revealed significant differences in the ΔT temperatures across the groups (F = 28.3 and *p* < 0.001), as shown in Figure 5 and Table 5.

Figure 5 displays the variation in delta temperature (ΔT) according to reflux classification, while Table 5 presents the results of Tukey’s post hoc test, which demonstrates the differences between the paired categories.

## 4. Discussion

This study sought to investigate the association between skin temperature and the severity of perforator veins (PV), validated through clinical examination and venous Doppler (VD). The primary findings reveal positive and significant associations between skin temperature and the classification of symptoms associated with the main PVs studied. Furthermore, a strong association was observed between elevated skin temperature in the studied regions of interest (ROIs) and PV dysfunction, with higher skin temperatures corresponding to more severe CEAP (Clinical, Etiological, Anatomical, and Pathophysiological) classification.

Accurate identification of PVs and their current state is pivotal in predicting the progression of venous diseases. To this end, we adopted a segmentation approach grouping the main PVs of the lower limbs into 30 areas, as previously reported by Caggiati et al. [6]. The increased thermal emission observed in the PV ROIs can be attributed to a thermodynamic mechanism that transfers the temperature from the deep venous system to the insufficient perforator system, therefore affecting the surrounding skin, as proposed by Soffer et al. [10].

Normal competent superficial veins do not exhibit significant differences on thermal imaging, making them challenging to detect on the skin’s surface. However, in cases of superficial venous disease, blood refluxes from the deep veins into the superficial veins through incompetent valves, particularly the great saphenous vein, causing heat to conduct to the surface. This heat is detected as an area of elevated skin temperature that corresponds to the course of the incompetent superficial vein, easily identifiable through infrared thermal imaging (IRTV).

In the present study, the maximum temperature (Tmax) recorded in the ROI for all studied venous perforators was consistently higher than 29 °C, with a peak of 35.65 °C (mean 32.17 ± 2.32 °C), as presented in Table 3. These findings align closely with those from previous studies, where skin temperatures were around 33 °C [10]. This alignment can be attributed to the dissipation of deep temperature (37 °C) as it reaches the superficial circulation, stabilizing at 29–30 °C, as discussed by Sanchez-Marin et al. [5]. Notably, our study, along with other investigations [10], identified an average temperature difference of 3.585 °C higher in cases of incompetent veins, indicating perforator dysfunction.

In the analysis of venous conditions, a significant difference in skin temperature was identified among patients with CEAP classifications of 2 and 4 (*p* = 0.014), as depicted in Figure 4. Notably, a more severe CEAP classification corresponded to a greater temperature difference (deltaT), ranging from a 1.8 °C increase for CEAP 2 to a 3.6 °C increase for CEAP 4. These findings are consistent with prior research indicating a close association between skin temperature and venous disease severity. However, it is important to note that not all studies have consistently reported such a strong relationship with the CEAP classification [18,19].

Although the presented study observed an association between thermographic parameters and CEAP classification, other researchers have described this association as relatively weak [19]. Consequently, the results suggest that although a connection exists between thermographic parameters and CEAP classification, it may not be clinically significant enough to effectively differentiate patients based solely on the CEAP classification, as noted by Kajewska et al. [19]. Despite its widespread adoption, the CEAP classification system has faced criticism, with concerns raised about its complexity, making it challenging to apply consistently; it lacks a functional assessment, focusing primarily on anatomical and clinical aspects; symptom evaluation can be subjective and vary among clinicians; it has limited predictive ability for clinical outcomes; it may exclude relevant factors such as quality of life assessments; it relies heavily on clinical appearance, potentially neglecting underlying pathophysiological factors, and it primarily focuses on lower-limb venous conditions.

Interestingly, alternative approaches have been proposed in these studies [18]. They propose that when diagnosing venous insufficiency using thermography, it may be prudent to establish a threshold for calculating the isotherm area based on the average limb temperature of healthy individuals within an appropriate age range rather than relying solely on the CEAP classification. This approach, involving the calculation of temperature differences (DT = Tmean, lesion − Tmean), has the potential to offer a valuable clinical practice, potentially providing a more accurate and clinically meaningful means of assessing CEAP classification for venous insufficiency through thermography [18].

Figure 5 showed significant differences in mean skin temperature delta between categories without reflux and those with mild reflux, supporting the association between delta T and reflux severity. Cut-off points were established based on delta T in relation to reflux severity (*p* < 0.001), resulting in the following average delta T values: no reflux (1.2 °C), mild reflux (1.8 °C), moderate reflux (2.9 °C), and severe reflux (3.6 °C), as outlined in Figure 5 and Table 5. These values provide a practical framework for categorizing the degree of venous reflux in perforator veins, though distinguishing between “moderate” and “severe” categories remains challenging. Recent studies have reported similar findings, and an analysis of the correlation between the range of reflux and the mean temperature of the affected area (CVD) according to Tmean CVD by Kajewska et al. [19] revealed a statistically significant moderate relationship with an R-value of 0.4 and a coefficient of 0.36, considering reflux duration.

Thermography offers the advantage of a non-invasive, holistic approach with immediate results, potentially aiding in early detection. In contrast, venous Doppler provides precise hemodynamic insights but may be operator-dependent and time-intensive. In the present study, venous Doppler consistently identified thermal alterations along the perforator vessel in all cases examined, manifesting as distinct hotspots contrasting with the surrounding tissue. This positions IRTV on par with venous Doppler in terms of sensitivity for detecting insufficient veins, albeit with a more functional approach than an anatomical one [10]. Other studies have reported similar agreements of greater than 90% between skin thermal increase in specific areas corresponding to PVs and those observed by VD. Thermal imaging can provide valuable insights into vascular anatomy and hotspots, supporting the early detection and assessment of relevant anatomical structures. It serves as a rapid and straightforward method for assessing cutaneous perforators in various anatomical regions, showing a high rate of confirmation when compared to hand-held Doppler [20]. This aligns with the present study’s assertion that thermography offers a more functional approach than an anatomical one, suggesting that thermal imaging can play a pivotal role in improving sensitivity for detecting insufficient veins, particularly in reducing examination time and enhancing the accuracy of perforator identification [21].

Thermal imaging (TI) exhibited high sensitivity and specificity, akin to the study by Xiao W et al., where they reported a sensitivity of 95% and specificity of 85% [22]. Currently, PV identification primarily relies on VD, which demonstrates a sensitivity of 94% for diagnosing varicose veins and 70% for detecting PVs, with sensitivity decreasing as the distance from the point of interest increases. However, the combined use of thermal imaging significantly increased the detection rates of small saphenous vein incompetence by 36% and trunk vein incompetence by 24.6% in the study by Soffer et al. [10]. Further analyses, including sensitivity and specificity calculations (ROC curve), are warranted to provide a comprehensive assessment of TI’s diagnostic performance.

Among the limitations of this study is the fact that it involved a limited number of participants and precluded a segmented analysis of each PV by the procedure operator. Consequently, only the most relevant PVs were studied. Additionally, this study relied on retrospective analysis, and we intend to lay the groundwork for future prospective and multicenter studies. Such an approach will facilitate a more robust assessment of IRVT’s potential in aiding the screening and monitoring of patients with insufficient perforator veins.

The primary contribution of this research lies in the innovative and promising use of IRTV for identifying insufficient perforator veins, offering a more accurate visualization of patients’ venous conditions. Thermography provides non-invasive assessment by visualizing skin temperature patterns, indicating underlying venous dysfunction. It allows for immediate results, portability, and a comprehensive view of the venous system, potentially enabling earlier detection of insufficiencies. However, it is crucial to acknowledge that diagnostic accuracy may be influenced by external factors such as ambient temperature and patient-related variables, potentially introducing variability [23].

On the other hand, VD offers a direct evaluation of blood flow dynamics, providing robust sensitivity and specificity in detecting venous insufficiencies. It excels in precisely localizing reflux sites and quantifying hemodynamic changes. Nevertheless, VD assessment can be operator-dependent, requiring skill and experience to obtain accurate results. The procedure may also be time-consuming and subject to anatomical variations, which might influence its practicability in routine clinical settings [24]. IRTV offers the advantage of providing a comprehensive view of the patient’s venous condition through a single image mapping the entire lower limb or a significant portion of it [10].

This aids in a broader and quicker assessment, guiding the use of VD and potentially enhancing its sensitivity in areas that might otherwise go unnoticed. Combining IRTV and VD presents unique advantages and limitations in the realm of identifying insufficient perforator veins, encompassing diagnostic accuracy and clinical practicability. In conclusion, our study supports the potential clinical utility of IRTV as a complementary tool to VD in diagnosing and monitoring venous insufficiencies, particularly in perforator veins. Further investigations, including larger prospective studies, are warranted to validate these findings and explore the full clinical potential of this approach.

The results of this study are relevant to healthcare practitioners, researchers, and patients alike. Healthcare providers may benefit from incorporating IRVT into their diagnostic toolkit, potentially enhancing their ability to detect and manage venous insufficiencies. Researchers can build upon these findings to advance the field of venous disease diagnosis further. Ultimately, patients stand to gain from earlier and more accurate diagnoses, potentially leading to improved quality of life and reduced healthcare costs. In summary, this study opens new avenues in the field of venous disease diagnosis by highlighting the promise of IRVT as an adjunct to traditional methods. However, it is crucial to recognize the study’s limitations and the need for more extensive research to fully realize its potential in clinical practice.

## 5. Conclusions

This study underscores the potential efficacy of dual infrared–visual thermography (IRVT) as a valuable adjunct to venous Doppler (VD) in identifying perforator veins (PV). IRVT, being non-invasive and reproducible, shows promise for early detection of insufficient PV. It also serves as a patient-friendly tool for discussing venous conditions. The study suggests the need for further research, incorporating larger datasets and leveraging temperature patterns, to refine CEAP classification and enable early identification of insufficient veins. Although these findings offer valuable insights, it is crucial to acknowledge the study’s limitations and emphasize the necessity for additional research to fully integrate IRVT into clinical practice.

## Figures and Tables

**Table 1:** Clinical-Etiology-Anatomy-Pathophysiology (CEAP) classification.

Clinic (C)	Etiology (E)	Anatomy (A)	Pathophysiology (P)
C0—No visible or palpable signs of venous disease	Ec Congenital	As veins superficial	Pr Reflux
C1—Presence of telangiectasias or reticular veins	Ep Primary	Ad veins deep	Po Obstruction
C2—Presence of truncal varicose veins C2r—Recurrent varicose veins	Es Secondary	Ap perforators system	Pro reflux and obstruction
C3—Swelling			Pn without identifiable cause
C4—Skin changes related to venous pathology: 4a: pigmentation, eczema 4b: lipodermatosclerosis, white atrophy 4c: crown phlebectatic			
C5—Skin changes + healed ulcer			
C6—Skin changes + active ulcer C6r—Recurrent ulcer			

**Table 2:** Results of thermographic evaluation of perforator veins in the lower limbs (*n* = 99).

Perforator	N	Tmax (°C)	SD	Tmin (°C)	SD	Delta T (°C)	SD
Boyd	13	33.05	2.071	29.96	2.433	2.18	0.773
Lower Cockett	3	31.33	0.751	29.50	1.253	1.83	1.115
Upper Cockett	3	31.37	1.935	29.93	0.808	1.43	1.286
Albanian Thigh	2	35.65	1.485	33.25	2.051	2.40	0.566
Dodd	2	34.15	1.202	32.35	2.192	1.90	0.990
Upper Fibular	12	32.73	2.206	31.17	2.545	1.57	0.768
Gemelar	1	32.60	-	28.80	-	3.80	-
Lower Gluteal	2	32.55	0.354	05.30	3.041	2.50	2.687
Hach	4	31.88	1.335	30.55	1.179	1.35	0.705
Hunter	21	32.48	2.577	30.20	2.703	2.29	1.138
Hunter-Dodd	1	34.50	-	30.60	-	3.90	-
Inguinal	1	29.60	-	26.10	-	3.50	-
Side knee	1	31.60	-	28.60	-	3.00	-
Kuster	1	29.80	-	28.70	-	1.10	-
May	3	32.53	3.062	27.63	4.225	4.50	0.900
Obturator	1	30.80	-	29.00	-	1.80	-
Tibial Bone	6	29.78	1.495	28.13	1.666	1.65	0.437
Poplitea	7	33.40	2.080	31.19	2.133	2.26	0.985
Sherman	15	31.48	2.052	29.71	2.446	1.82	0.927

SD: Standard Deviation; Boyd: medial malleolar vein; Lower Cockett: posterior tibial vein; Upper Cockett: anterior tibial vein; Albanian Thigh: perforators veins of the thigh; Dodd: peroneal vein; Upper fibular: uppermost perforator of the peroneal vein; Gemelar: perforators veins of the calf; Inferior Gluteal: inferior gluteal vein; Hach: perforator located in the upper thigh; Hunter: perforators veins of the thigh and leg; Hunter-Dodd: junction between Hunter and Dodd perforators; Inguinal: inguinal vein; Side knee: lateral knee perforators; Kuster: perforator located at the medial ankle; May: perforators vein of the medial calf; Obturator: obturator vein; Tibial Bone: perforator located at the medial malleolus; Poplitea: popliteal vein; Sherman: lateral perforators of the lower leg.

**Table 3:** Results of thermographic evaluation according to CEAP classification of veins in lower limbs (*n* = 99).

CEAP	N°	Tmax (°C)	SD	Tmin (°C)	SD	Delta T (°C)	SD
(1–6)							
1	1	33.50	-	31.80	-	1.70	-
2	12	31.95	1.530	30.73	1.645	1.38	0.536
3	33	31.73	2.344	29.32	2.715	1.91	1.132
4	37	32.86	2.081	30.48	2.230	2.45	1.138
5	2	32.20	2.828	29.40	1.697	2.80	1.131
6	14	32.26	2.817	30.16	2.982	2.15	0.829

CEAP = Clinical-Etiological-Anatomical-Pathophysiological classification system; Tmax = maximum temperature; Tmin = minimum temperature; delta T °C = difference between Tmax and Tmin (in degrees Celsius).

**Table 4:** Results of the thermographic evaluation according to the classification of venous reflux in the lower limbs (*n* = 99).

	Reflux			Confidence Interval at 95%			Shapiro–Wilk	
		No	Average	Lim. Bottom	Lim. Higher	Standard Deviation	W	*p*
Tmax °C	No reflux	24	31.56	30.656	32.47	2.148	0.976	0.817
	Mild	41	32.03	31.362	32.70	2.122	0.965	0.227
	Moderate	27	32.33	32.169	2.34	2.335	0.971	0.619
	Severe	7	33.10	31.079	12.35	2.186	0.986	0.984
Tmin °C	No reflux	24	29.82	28.777	30.87	2.482	0.944	0.204
	Mild	41	30.28	29.544	02.31	2.332	0.976	0.532
	Moderate	27	30.13	29.039	31.21	2.749	0.968	0.554
	Severe	7	29.46	27.201	31.71	2.439	0.980	0.959
Delta T °C	No reflux	24	1.20	0.981	1.43	0.530	0.925	0.077
	Mild	41	1.83	1.590	2.08	0.774	0.967	0.269
	Moderate	27	2.91	2.544	3.27	0.920	0.964	0.451
	Severe	7	3.59	2.612	4.56	1.053	0.874	0.200

Lim.: Limits; W: Shapiro–Wilk statistics; *p*: *p*-value from Shapiro–Wilk test.

**Table 5:** *p*-value results from Tukey’s post hoc test for temperature deltas (ΔT), indicating differences between pairs (*n* = 99).

	Mild Reflux	Moderate Reflux	Severe Reflux
No reflux	0.013 *	<0.001 ***	<0.001 ***
Mild reflux	—	<0.001 ***	<0.001 ***
Moderate reflux		—	0.185

* *p* < 0.05, *** *p* < 0.001.

## Data Availability

Data not available due to privacy or ethical constraints.
